# Temporomandibular Disorders Management—What’s New? A Scoping Review

**DOI:** 10.3390/dj12060157

**Published:** 2024-05-23

**Authors:** Giovanni Mauro, Alessio Verdecchia, Carlota Suárez-Fernández, Riccardo Nocini, Enrico Mauro, Nicoletta Zerman

**Affiliations:** 1 Department of Medicine and Surgery, Dental Clinic, University of Parma, 43125 Parma, Italy; giovannimauro@mac.com; 2 Department of Surgical Sciences, Postgraduate School in Orthodontics, University of Cagliari, 09124 Cagliari, Italy; 3 Department of Surgery and Medical-Surgical Specialities, School of Medicine and Health Sciences, University of Oviedo, 33003 Oviedo, Spain; suarezcarlota@uniovi.es; 4 Department of Surgical Science, Dentistry, Pediatrics and Gynecology, University of Verona, 37134 Verona, Italy; riccardo.nocini@univr.it; 5 Faculty of Medicine, University of Milano, 20122 Milano, Italy; mauroenrico@me.com; 6 Pediatric Dentistry and Oral Hygiene Unit, IRCCS Sacro Cuore-Don Calabria Hospital, 37024 Negrar di Valpolicella, Italy

**Keywords:** temporomandibular joint disorders, therapeutics, review

## Abstract

Temporomandibular disorders include various conditions that impact the masticatory system, affecting its structure, function, or physiology. Clinicians face a complex array of therapeutic options when treating this group of diseases, emphasizing the importance of evaluating the current evidence to guide decisions in patient care. The main objective of this article is to conduct a scoping review on the available treatment approaches to manage temporomandibular disorders (TMDs). An extensive search of the literature was performed on Scopus, Pubmed, Embase, and Web of Science. Systematic reviews published in the last 5 years were considered. Out of the 2183 publications identified, 109 studies were included in this review. Among them, 39 articles focused on the non-invasive approach, while 120 delved into the minimally invasive approach, and 15 explored the surgical approach. Non-invasive or conservative approaches like cognitive-behavioral therapy, physical therapy, and acupuncture offer effective pain management and functional improvements in TMDs. Emerging treatments offer promising alternatives for treating these disorders. Surgery should be reserved for severe cases, with conservative therapies used in conjunction with invasive procedures for optimal patient outcomes.

## 1. Introduction

Temporomandibular disorders (TMDs) encompass a range of diseases or disorders that impact the structure, function, or physiology of the masticatory system. TMDs primarily include disorders associated with pain, such as myalgia, myofascial pain, or arthralgia, as well as disorders related to functional or structural changes in the temporomandibular joint, such as disorders of the disc and degenerative joint conditions [1].

The prevalence of these disorders varies depending on the age group, with adults and the elderly presenting a prevalence of 31%, while children and adolescents have a prevalence that varies between 20% and 60%. Additionally, the most common disorder is disk displacement with reduction [2,3]. Based on recent epidemiological findings and scientific evidence, it has been noted that women have twice the risk of developing TMDs compared to men [4]. Gender differences have also been observed in pain tolerance and expectations; in temporal summation and somatic perception, female gender is also the most significant risk factor [5].

The etiology is multifactorial and there is no evidence of a link between genetic variants and TMDs [6]. The main factors associated with this group of disorders are third molar extraction, orthognathic surgery, catastrophizing, and bruxism [7,8,9,10]. TMDs also show a comorbid relationship with other diseases or risk factors, which increase the rate of their development, such as systemic rheumatic disease; psychosocial factors such as somatization, depression, and distress; and Parkinson’s disease [11,12,13].

The diagnosis of TMDs involves a comprehensive assessment that includes a detailed history, clinical examination, and imaging. However, the goal of TMD assessments is to identify one or more biomarkers (molecules such as prostaglandin (PG), matrix metalloproteinases (MMPs), interleukins (ILs), tumor necrosis factor (TFN), bradykinin, glutamate, neuropeptides, nerve growth factor (NFG), and serotonin; neuroimaging markers such as N-acetyl aspartate (NAA), choline (Cho), total creatine (tCr), glutamine (Gln), and myo-insitol; and biochemicals such as vitamin D and 8-hydroxydeoxyguanosine and malondialdehyde) that are involved in the manifestation of painful TMDs [14]. TMDs can manifest a variety of clinical manifestations that can only emerge through a very accurate history. They are often associated with sleep disturbances such as obstructive sleep apnea and gastroesophageal reflux; cervical musculoskeletal disorders and fibromyalgia; primary headaches; and tinnitus [15,16,17,18,19,20]. Depending on the magnitude of the TMD, the quality of life can also deteriorate [21,22].

Regarding treatment approaches for TMDs, clinicians face a complex array of therapeutic options, and recent research findings have not simplified this dilemma but rather added to the complexity. The modern management of temporomandibular disorders (TMDs) is based on a biopsychosocial model and an orthopedic approach, as they are considered as a musculoskeletal group of diseases rather than solely a dental or occlusal problem [23].

Evidence-based medicine (EBM) involves the careful and deliberate utilization of the current best evidence to guide decisions in patient care [24]. Given the multidisciplinary nature, the variety of available treatment approaches, and the emergence of new therapies for TMDs, it is imperative to synthesize the current evidence to enhance clinical practices. Therefore, the aim of this article is to provide a scoping review of the treatment strategies available to manage TMDs.

## 2. Materials and Methods

This scoping review was performed in accordance with the Preferred Reporting Items for Systematic Reviews and Meta-Analyses extension for conducting scoping reviews (PRISMA-ScR) [25]. This scoping review was not registered.

### 2.1. Search Strategy

A systematic search was conducted on Scopus, PubMed, Embase, and Web of Science. The search strategy designed for each database is shown in Table 1.

The inclusion criteria were systematic reviews and meta-analyses with evidence-based methodologies for the assessment and evaluation of findings. Papers that made claims about the efficacy of devices, diagnostic tools, or therapeutic methods without evidence-based research were excluded.

### 2.2. Data Extraction and Synthesis

The review process initially involved screening the titles and abstracts. Then, the full text of the selected papers was assessed. Two different reviewers (GM, NZ) independently conducted the process and discordant evaluations were resolved through a discussion with a third researcher (AV). The final decision on inclusion or exclusion in the review was reached through consensus between all the authors. The full text of each study was retrieved and independently evaluated by two authors. After thorough discussion, the studies were categorized into the following classes: diagnosis, clinical implications, treatment, and prevention.

For each article, we extracted the following information: author, year of publication, country of the primary author’s institution, number and type of studies included in the review, treatment approaches evaluated, and conclusions.

## 3. Results

### 3.1. Literature Screening Process

**Figure 1:**
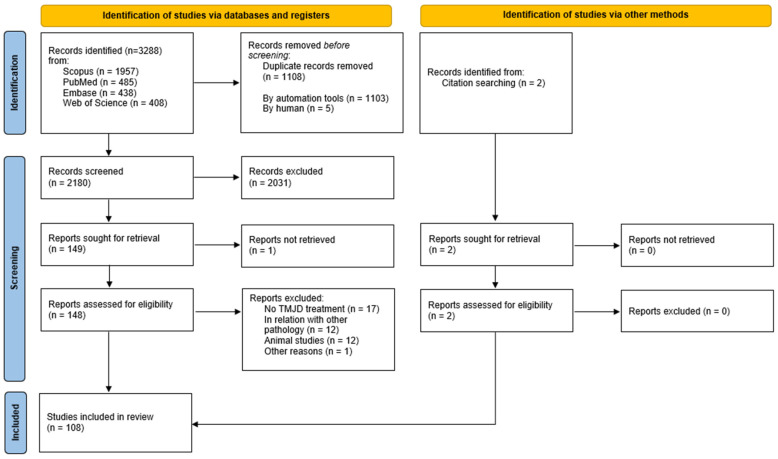
Flowchart of study selection process [26].

The search strategy yielded a total of 3288 publications, including 1957 from Scopus, 485 from Pubmed, 438 from Embase, and 408 from Web of Science. By removing duplicates, 1108 publications were excluded. From the remaining 2180 records, after reviewing the title and abstract, 2031 studies were excluded. Afterwards, the full text of the remaining 148 studies was thoroughly examined; one study could not be retrieved. After assessment of the full text, 42 reports were excluded. Ultimately, 106 records met the inclusion criteria. Additionally, two publications were retrieved by citation searching. The literature search and screening procedure are detailed in Figure 1.

### 3.2. Description of the Selected Studies

Based on the treatment assessed, the articles were categorized into non-invasive, minimally invasive, and invasive (Table 2).

#### 3.2.1. Study Characteristics

The countries with the highest number of reviews were China (n = 14) [47,57,59,63,70,76,77,79,104,105,112,114,122,124], followed by Brazil (n = 13) [33,44,64,90,93,106,107,111,115,123,127,129,131], and Spain (n = 9) [36,38,41,43,45,52,68,103,110]. The number of studies encompassed in these reviews ranged from 52 [34,52,125] to 3 [102], with RCTs predominating the dataset. All the characteristics of the included studies are documented in Table 3.

#### 3.2.2. Treatment Approaches

The treatment approaches were classified into three categories: non-invasive (conservative), minimally invasive, and invasive.

##### Non-Invasive (Conservative) Approaches

- Cognitive–behavioral therapy (CBT) and counseling

CBT has been widely acknowledged as efficacious in the management of orofacial pain (OFP), as well as in mitigating psychological distress stemming from TMDs [27]. Counseling is also a valuable addition to traditional conservative approaches like splints or manual therapy [29]. However, a recent Cochrane review (2022) found only limited evidence that CBT has a greater capacity to diminish pain intensity compared to alternative treatments or control groups, but not at treatment completion [27].

- Physical therapy, MT, and exercises

A systematic review indicated that either a mobility or mixed approach may alleviate discomfort and enhance mobility, but with a limited impact on functional improvement [41]. MT and therapeutic exercise stand as efficacious therapeutic modalities for diminishing pain, elevating pain pressure thresholds, and augmenting both active and passive MMO in individuals afflicted with TMDs. They may be beneficial and play a role in the treatment of disc displacement without reduction [43,52]. Oral myofunctional therapy has also been effective in reducing the pain intensity compared to other conservative treatments [53]. Investigations indicated the potential utility of physiotherapy as an advantageous therapeutic avenue for individuals with TMJ dysfunction. Nonetheless, evaluating the efficacy of distinct therapeutic modalities for TMJ patients and discerning whether particular TMJ pathologies exhibit a heightened responsiveness to conservative management could furnish valuable insights into the effectiveness of conventional interventions within this patient cohort [34].

Physical therapy interventions have shown benefits for TMD patients with comorbid headaches [32].

MT has exhibited efficacy as an intervention for temporomandibular disorders in the intermediate duration, albeit with a diminishing impact observed longitudinally. Nevertheless, the integration of MT with therapeutic exercise has the potential to sustain these therapeutic outcomes over the extended term [41].

Regarding comparisons, MT has demonstrated superiority over an absence of treatment in one investigation and outperformed counseling in another. However, when MT is amalgamated with counseling, it does not exhibit statistically significant superiority over counseling in isolation. Furthermore, MT in isolation did not produce better results when contrasted with the effects of botulinum toxin. In a separate study, the combination of MT with home-based therapy proved more efficacious than home-based therapy alone. Nevertheless, due to inconclusive data and inadequate homogeneity, further research is warranted to offer more conclusive determinations [33].

Concerning cervical MT interventions, the findings indicated their superior efficacy in diminishing the pain intensity compared to placebo MT or minimal intervention, which is bolstered by moderate evidential support. Combined cervico-craniomandibular interventions yielded greater short-term reductions in pain intensity and enhanced pain-free maximum mouth opening in patients afflicted with TMDs and headaches, although the inconclusive evidence hampers drawing definitive conclusions [36]. The application of cervical-mandibular manual therapies, alongside exercise and educational components, has exhibited superior outcomes compared to exercise/education alone in individuals experiencing tinnitus attributed to a TMD [118].

In order to ascertain the optimal manual therapy methodologies for alleviating pain and enhancing maximum mouth opening in individuals with TMDs, high-caliber research employing diverse techniques across various regions and patient demographics is imperative [39].

Yet another systematic review incorporating a meta-analysis indicated that rehabilitative strategies could potentially surpass placebo and sham interventions in mitigating pain among individuals with muscle-related TMDs. Nonetheless, the scarcity of randomized controlled trials appraising conservative methodologies impedes the amalgamation of the evidence concerning distinct techniques, underscoring the necessity for circumspection in interpreting these findings [42].

- Other options

Numerous new treatment options are continuously being introduced, but only a few have sufficient supporting evidence.

Oxygen–ozone (O2O3) therapy exhibited promise in reducing TMD pain and enhancing MMO, especially when employed during arthrocentesis. Nonetheless, there is a dearth of conclusive evidence regarding its superiority compared to occlusal splints and pharmacological interventions. The studies included in this review exhibited considerable diversity in terms of comparators, application techniques, ozone concentration, treatment frequencies, and follow-up durations. Further double-blind clinical trials are imperative to consolidate our understanding before contemplating the integration of OT into clinical TMD management protocols [55]. In most studies, ozone therapy is placed in the context of a mixed approach as an adjunct to other conservative or minimally invasive treatments for TMDs [34,40,54,56].

In the systematic reviews analyzed, only one article mentions ultrasonic therapy, comparing it to acupuncture and affirming the latter technique’s greater therapeutic effects on TMDs than the former [57].

##### Minimally Invasive Approaches

- Arthrocentesis or intra-articular injections

It can be seen inferred that TMJ arthrocentesis enhances mandibular functionality and alleviates the pain intensity, with multiple sessions (ranging from three to five) exhibiting greater efficacy compared to a solitary session [58]. The limited data suggest that there is no notable disparity in pain or maximal mandibular aperture between single- or double-puncture techniques for arthrocentesis [61].

Intra-articular CCS or NSAID analgesic injections do not seem to provide additional advantages over lavage [57]. On the other hand, injections of hyaluronic acid (HA) administered within the joint have shown benefits in improving the functional symptoms and pain associated with TMDs [65]. Still, the evidence suggested that intra-articular pharmacological injections of corticosteroids, hyaluronic acid, and platelet-rich plasma did not produce any significant improvement in temporomandibular joint pain and functional outcomes when compared with placebo injections [78].

A systematic review incorporating a meta-analysis examining the comparative risk profiles associated with arthroscopy and arthrocentesis procedures of the TMJ determined that there is no elevated likelihood of complications with arthroscopy vis à vis arthrocentesis. Moreover, the complications observed were transient in nature [63].

Yet another systematic review evaluated the effectiveness of splint therapy in enhancing outcomes subsequent to arthrocentesis, encompassing six investigations. This review revealed no statistically notable discrepancy in pain mitigation or enhancement of the maximal mouth aperture, whether splint usage was involved or not following arthrocentesis, both at one month and six months [68]. The ideal timing for conducting arthrocentesis in TMD management was assessed in a review encompassing eight randomized controlled trials and three prospective clinical investigations. Whether performed early or late as the initial intervention, arthrocentesis exhibited enhancements in mouth opening and pain alleviation [72].

Moreover, numerous novel compounds have undergone scrutiny regarding their efficacy in managing TMJ pain and augmenting mandibular abduction. These encompass analgesic agents, dextrose coupled with lidocaine, adipose tissue, hematopoietic stem cells, and ozone. Administering arthrocentesis before the injection appears to augment the efficacy of intra-articular delivery, with bone marrow and adipose tissue showing the most promising results [85]. Among individuals diagnosed with temporomandibular joint osteoarthritis (TMJ-OA), arthrocentesis has demonstrated efficacy in significantly alleviating pain and enhancing mandibular functionality. However, additional injections of hyaluronic acid (HA), either low-molecular-weight (LMW) HA or high-molecular-weight (HMW) HA, or cortisone at the end of arthrocentesis did not lead to further improvements in clinical outcomes [97]. A systematic review comparing the treatment of TMJ-OA with HA, corticosteroids, and blood products in conjunction with arthrocentesis found that all substances efficiently alleviated pain and improved MMO [80]. For TMJ-OA, tramadol, morphine, and platelet-derived growth factor (PDGF) injections after arthrocentesis have shown positive effects in reducing pain and improving joint opening, and short-term improvements in maximal mouth opening have also been observed with hyaluronic acid injections in TMJ-OA patients [69]. In cases of recurrent TMJ luxation, intra-articular autologous blood injection combined with pericapsular tissue application, accompanied by intermaxillary fixation, stands out as the most scientifically substantiated treatment approach. Nonetheless, methodologically robust studies, encompassing adequate patient cohorts, extended follow-up periods, and comprehensive patient-reported outcome measures, are required to delineate the optimal surgical therapeutic modalities [73].

- PRP, PRF, PRGF, PDGF, and stem cell therapy

An expanding corpus of research indicates the prospective advantages of intra-articular PRP, PRF, PRGF, and PDGF injections in managing TMDs. According to the current evidence, PRP injections may provide a greater pain reduction compared to placebo injections in temporomandibular joint osteoarthritis (TMJ-OA) at both 6 months (moderate level of evidence) and 12 months (moderate level of evidence) following the injection [76]. PRP and PRF exhibited similar short-term efficacies in treating TMDs, while PRF was more advantageous in terms of long-term efficacy. Therefore, PRF was recommended for treating TMDs [75]. Moreover, in comparison to saline, PRP exhibits a prolonged duration of pain reduction and augmentation of MMO. Nevertheless, further standardized RCTs are imperative to address the discrepancies in preparation protocols and study heterogeneity across different groups [86].

PRP injections provided adjuvant efficacy to arthrocentesis or arthroscopy in pain reduction for temporomandibular joint osteoarthritis in the long term. Furthermore, PRP injections significantly reduced pain better than HA injections, saline injections, or no injections [79].

In a systematic review assessing the benefits of applying PRP or PRGF injections simultaneously or after arthrocentesis or arthroscopy, eight randomized controlled clinical trials were analyzed. The utilization of intra-articular injections of PRP and plasma rich in growth factors (PRGF) showcased noteworthy distinctions in pain alleviation across three investigations, along with enhanced mandibular function, evidenced in two studies [67].

Based on limited evidence, the intra-articular introduction of mesenchymal stem cells into the TMJ could potentially yield significant effectiveness in diminishing joint pain and enhancing MMO in individuals with TMDs [83].

- Acupuncture

Although some reviews claim that the current evidence on acupuncture is limited regarding treatments for TMDs [87], other evidence supports exactly the opposite, stating that acupuncture promotes an improvement in TMDs and reduces pain [88]. Some authors claimed that some variants of acupuncture, such as that performed with a hot needle, have superior effects compared to traditional acupuncture, ultrasonic therapies, laser acupuncture, and drugs in the treatment of TMDs [57].

Laser acupuncture has also shown promise in relieving the signs and symptoms of TMDs when combined with traditional acupuncture and an occlusal splint [89].

- Botulinum toxin

Several reviews have examined the potential use of botulinum toxin (BTX) for various orofacial pain conditions. For instance, BTX has demonstrated effectiveness in treating refractory myofascial pain related to TMDs and bruxism [94]. However, the evidence regarding the effectiveness of BTX in managing TMDs and bruxism is currently not fully conclusive. Nevertheless, several studies meeting the inclusion criteria have reported promising findings, underscoring the need for further investigation [35,41,91].

- Drugs

Pharmacological agents commonly employed in TMD treatments encompass NSAIDs, opioids, CCS, muscle relaxants, antidepressants, anticonvulsants, and benzodiazepines [28,47,62,69,82,84,85,98,99,100,101,102]. Regarding pain management in TMDs, some evidence suggests that NSAIDs can be considered as an initial approach for alleviating joint and muscle pain in TMD patients [99,100]. Although some authors have stated that depending on the origin of the pain associated with TMDs, the pharmacological choice is different. For TMDs of muscular origin, the best results were obtained with BTX-A, granisetron, PRP, and muscle relaxants, while for TMDs of a joint nature, the most effective treatments were NSAIDs, CCS, HA, and dextrose [82].

- Laser and TENS

Multiple systematic reviews have indicated that LLLT is an effective method for pain relief and improvement of functional outcomes in patients with TMDs, including both artrogenous and myogenous conditions [104,107,111,112]. While TENS leads to decreased electrical muscle activity in the masticatory muscles, a reduced masseter muscle thickness, enhanced functionality and comfort in daily activities, and alleviated pain linked to temporomandibular disorders (TMDs), the scientific evidence supporting these effects appears to be of moderate quality [109,110]. The findings of the meta-analysis revealed that LLLT exhibited superior short-term effectiveness compared to TENS in addressing TMD pain. Enhanced outcomes can be attained with increased wavelengths. Consequently, we advocate for the utilization of LLLT with wavelengths ranging from 910 nm to 1100 nm for treating TMDs [103].

- Oral splints

Occlusal splint therapy has not been proven to provide any additional benefit in TMDs compared to standard modalities [121]. Multiple studies have shown that occlusal splinting alone or combined with other therapeutic modalities is the most effective treatment option for reducing TMD pain in the short term [29,122,123]. Some reviews have compared splinting with other therapeutic modalities and stated that therapeutic exercises have not shown a clear superiority over occlusal splints for the treatment of painful TMDs [121], and that the use of a splint therapy does not improve the effects of arthrocentesis [116]. It is worth noting that positive findings emerge when the Diagnostic Criteria for Temporomandibular Disorders (DC/TMD) are applied, highlighting the need for standardization in diagnosing and managing TMDs [123]. Despite the treatment results of stabilization appliances, the improvements observed may be attributed to a placebo effect [116]. All types of occlusal splints, such as the anterior repositioning splint, hard stabilization splint, soft stabilization splint, mini anterior splint, and prefabricated splint, are likely to be more effective treatments for arthrogenous and myogenous temporomandibular disorders (TMDs) when compared to receiving no treatment (untreated control patients) or using non-occluding splints. Regarding patients primarily experiencing arthrogenous TMDs, limited-quality evidence suggests that the anterior repositioning splint and counseling therapy in conjunction with a hard stabilization splint are the most effective treatments for reducing pain and temporomandibular joint (TMJ) sounds. In cases of mainly myogenous TMDs, there is a very low level of evidence suggesting that mini anterior splints may offer the most effective treatment in reducing subjective pain outcomes [31].

An examination of the impacts of occlusal splints on enhancements in spinal posture among patients with TMDs implies that occlusal splints may represent a non-invasive therapeutic modality for TMD management. Nevertheless, owing to the scarcity of robust studies in this domain, additional investigations employing combined force platform stabilometry and kinematic evaluation of spinal dynamics are imperative to elucidate the influence of occlusal splints on posture [40].

In conclusion, it is important to note that while all these minimally invasive approaches show promise, further research, particularly standardized RCTs, is necessary to establish their efficacy, optimize the techniques, and determine their long-term effects.

##### Surgical Procedures

Among the 15 articles exploring surgical approaches, 7 examined minimally invasive surgery, while 8 focused on open surgery.

One of the latest studies suggests that arthroscopic surgery should be performed on masses confined to the superior TMJ space, while open arthroplasty is indicated in cases with extra-articular extension. A combination of both treatment methods may be necessary when the lesion extends beyond the medial sulcus of the condyle [131].

A systematic review comparing various surgical techniques, including gap arthroplasty (GA), interpositional gap arthroplasty (IGA), reconstruction arthroplasty (RA), and distraction osteogenesis (DO), revealed that IGA with autogenous materials, along with reconstruction employing autologous grafts or total joint replacement using alloplastic prosthetic implants, demonstrate comparable clinical results in the treatment of ankylosis [132].

Other systematic reviews assessing different total temporomandibular joint prosthesis systems demonstrated significant improvements in both preoperative and postoperative outcomes, with no notable differences observed between the various devices [126,127].

A systematic review comparing minimally invasive procedures with invasive surgical techniques for artrogenous TMJ management revealed lower VAS scores and higher maximum incisal opening (MIO) values after discectomies and discoplasties in the within-group comparison after discectomy [128].

However, the current scientific evidence remains unclear, and invasive surgical procedures should not be regarded as an effective primary treatment modality for arthrogenous temporomandibular disorder TMD management, notwithstanding the lower VAS scores and elevated MIO values noted post-discectomy in contrast to arthroscopy, eminectomy, and discoplasty.

Overall, while there are various surgical options available for TMJ ankylosis and other TMD conditions, it is crucial to carefully consider their use and prioritize less invasive approaches before resorting to surgery.

## 4. Discussion

This scoping review provides an in-depth analysis of the most recent scientific evidence regarding the therapeutic approaches for temporomandibular disorders (TMDs). Through a thorough examination of the literature, it became evident that these approaches are mainly divided into conservative or non-invasive, minimally invasive, and invasive treatments, with a wide range of specific therapeutic options available within each category.

Chronic pain remains a major concern of TMDs, and its management is a primary focus for healthcare professionals. According to some studies, such pain would appear to be related to individual psychological profiles and the pain application status [134,135]. Biological, psychological, and social factors interact with contextual and environmental stressors, generating painful TMDs and associated symptoms [136]. The existing evidence supports a positive association between work-related stress and temporomandibular disorders (TMDs), highlighting the need for primary prevention interventions [62,137]. Addressing stress in the workplace is crucial to preventing the development or worsening of TMDs. However, advancements in neuroimaging techniques have provided valuable insights into the underlying neuro-pathophysiological mechanisms involved in TMDs. These techniques, such as magnetic resonance imaging (MRI), have improved the understanding of the structural and functional alterations within the temporomandibular joint and surrounding tissues. This improved understanding helps inform treatment decisions and facilitates more targeted interventions [20].

In addition to pain management, subjective sleep quality has emerged as an important consideration in the management of TMDs. Sleep disturbances are frequently experienced by individuals with TMDs and have the potential to worsen their symptoms [15,138,139]. Addressing sleep quality through appropriate interventions, such as sleep hygiene practices or targeted treatments for sleep disorders, can have a positive impact on TMD outcomes.

In agreement with similar studies, the treatment goals for TMD encompass various aspects, including pain control, improved mandibular function, and the restoration of normal daily activities [140,141]. A multidisciplinary approach is often employed, incorporating conservative modalities such as home care regimens (e.g., self-care exercises and relaxation techniques), intraoral appliance therapy (e.g., splints or orthotics), physiotherapy, pharmacotherapy, local anesthetic trigger point injections, and complementary modalities (e.g., acupuncture or low-level laser therapy). These interventions are targeted at mitigating pain, enhancing functionality, and augmenting the overall well-being of these patients. In terms of diagnostic tools, clinical guidelines are frequently employed as initial screening aids for TMDs. These protocols help identify potential TMD cases and determine the need for further diagnostic investigations, such as imaging studies like MRI. However, there remains a lack of standardized diagnostic criteria across studies, leading to inconsistencies in TMD diagnoses. This inconsistency poses challenges when comparing findings and outcomes between different research studies.

Inherent to the design of scoping reviews, our study presents several limitations. As a result, our primary aim was to offer a comprehensive overview rather than an in-depth analysis of the information on TMD management. Furthermore, we did not conducted a risk bias assessment or meta-analysis due to the heterogeneity of the studies. Additionally, by restricting our inclusion criteria to studies published in English and from 2017 onwards, there is a potential risk of excluding significant research that was published before 2017 or in other languages. Furthermore, despite the inclusion of four databases, it may have been prudent to also include Google Scholar to mitigate any biases resulting from missing articles.

Overall, while advancements have been made in understanding and managing TMDs, there is still a need for well-conducted studies that employ established diagnostic parameters and outcome measures. This will contribute to a more comprehensive and reliable body of evidence, allowing for better comparisons and evidence-based guidelines for TMD management. The ongoing efforts to address these research gaps will help improve the care and outcomes for TMD patients in the future.

## 5. Conclusions

In conclusion, a multidisciplinary strategy is favored over singular therapies.

- Initially, non-invasive methods such as cognitive–behavioral therapy, physical therapy, and exercises should be prioritized. If these approaches are not effective, minimally invasive treatments like arthrocentesis and intra-articular injections may be considered.
- Surgery should be reserved for severe cases, with conservative therapies used in conjunction with invasive procedures for optimal patient outcomes.
- Furthermore, there is a need for standardization and higher-quality research to further advance the field. Clinicians should stay updated on the latest findings and prioritize preventive measures to reduce the chronicity of TMDs.

## Figures and Tables

**Table 1:** Search strategy for Scopus, PubMed, Embase, and Web of Science.

Database	Search Strategy	Number of Results
Scopus	(TITLE-ABS-KEY (“temporomandibular joint disorder”) OR TITLE-ABS-KEY(“temporomandibular joint disease”) AND TITLE-ABS-KEY(“treatment”)) AND PUBYEAR > 2018 AND PUBYEAR < 2025	1957
PubMed	((“temporomandibular joint disorder” [MeSH Terms] OR (“temporomandibular” [All Fields] AND “joint disease” [MeSH Terms])) AND “therapeutics” [MeSH Terms]) AND (2019:2024[pdat])	485
Embase	(‘temporomandibular joint disorder’: ti,ab,kw OR ‘temporomandibular joint disease’: ti,ab,kw) AND ‘treatment’: ti,ab,kw AND [2019–2024]/py	438
Web of Science	((TS = (“temporomandibular joint disorder ”)) OR TS = (“temporomandibular joint disease ”)) AND TS = (“treatment”)	408

**Table 2:** Treatment approaches and corresponding references.

Treatment		References
Non-invasive approach (n = 39)	Cognitive–behavioral therapy and counseling (n = 8)	[27,28,29,30,31,32,33,34]
	Physical therapy, manual therapy, and exercises (n = 25)	[28,29,30,32,33,34,35,36,37,38,39,40,41,42,43,44,45,46,47,48,49,50,51,52,53]
	Oxygen–ozone (O2O3) therapy (n = 5)	[34,40,54,55,56]
	Ultrasonic therapy (n = 1)	[57]
Minimally invasive approach (n = 120)	Arthrocentesis and Intra-articular injections (n = 23)	[28,38,43,48,51,58,59,60,61,62,63,64,65,66,67,68,69,70,71,72,73,74]
	PRP, PRF, PRGF, PDGF, and stem cell therapy (n = 23)	[48,51,54,56,62,64,66,67,69,70,73,75,76,77,78,79,80,81,82,83,84,85,86]
	Acupuncture (n = 8)	[47,50,54,57,87,88,89,90]
	Botulinum toxin (n = 13)	[29,34,35,47,82,91,92,93,94,95,96]
	Drugs (n = 13)	[28,47,56,62,69,82,84,97,98,99,100,101,102]
	Laser and transcutaneous electric nerve stimulation (n = 18)	[29,40,47,48,50,103,104,105,106,107,108,109,110,111,112,113,114,115]
	Oral splints (n = 22)	[28,29,30,31,40,42,47,48,49,51,62,68,116,117,118,119,120,121,122,123,124,125]
Surgical approach (n = 15)	Minimally invasive surgery (arthroscopy) (n = 7)	[51,60,63,66,67,83]
	Open surgery (n = 8)	[126,127,128,129,130,131,132,133]

**Table 3:** Characteristics of included systematic reviews and meta-analyses.

Author (Year)	Country	Number and Type of Studies Included	Treatment Approaches Evaluated	Conclusions
Thorpe ARDS et al. (2023) [28]	Australia	7 RCTs	Arthrocentesis vs. occlusal splint therapy, MT, therapeutic exercise, NSAIDs, CBT	Arthrocentesis > conservative management in
				- Reducing pain; - Improving jaw opening.
Patel J et al. (2019) [35]	United Kingdom	11 CTs	BTX	BTX should be considered but due to financial implications and possible side effects, conservative options, such as self-management with explanation and physical therapies, should be exhausted first.
Kelemen K et al. (2024) [30]	Hungary	10 RCTs	Comparison between splint therapy along with physiotherapy, manual therapy, and counseling vs. physiotherapy, manual therapy, and counseling alone	Combination therapy and physiotherapy for myogenic TMDs. Combination therapy did not prove superior to physiotherapy alone, casting doubt on the need for additional splint therapy.
Guarda-Nardini L et al. (2021) [58]	Italy	30 RCTs	Arthrocentesis	TMJ arthrocentesis ↑ jaw function and ↓ pain levels. Multiple sessions (3–5) > single session.
Hu Y et al. (2023) [59]	China	8 CTs	Arthrocentesis vs. conservative management	TMJ arthrocentesis: small improvement in pain scores without any improvement in MMO vs. conservative therapies.
Tang YH et al. (2024) [60]	The Netherlands	13 CTs	Arthroscopy vs. arthrocentesis vs. conservative treatments	TMJ arthroscopy: Similar pain reduction and complication rates to arthrocentesis. Arthroscopic lysis and lavage is superior to arthrocentesis in ↑ MMO at intermediate-term follow-up.
Chęciński M et al. (2023) [85]	Poland	22 RCTs	Injection of autologous blood	Injections of autologous blood were effective in preventing further TMJ dislocation episodes in 75–94% of patients. Mouth opening was reduced by 10–20% and the articular pain ↓. No cases of post-interventional ankylosis were identified.
Chęciński M et al. (2022) [83]	Poland	5 CTs	Autologous stem cell transplants	IA administration of mesenchymal stem cells to TMJ (based on weak evidence) ↓ articular pain and ↑ MMO in TMDs.
Thambar S et al. (2020) [91]	Australia	7 RCTs	BTX	Despite showing benefits, consensus on the therapeutic benefit of BTX in the management of myofascial TMD is lacking.
Machado D et al. (2020) [92]	Brazil	12 RCTs	BTX-A	- BTX-A is well-tolerated and produces a slight improvement in painful temporomandibular disorders vs. placebo at one month but not at three or six months. - Other active treatments (occlusal plates, behavioral interventions, medication) and low-level laser at 1, 6, and 12 months were tested.
Marliere DAA et al. (2023) [130]	Brazil	11 CTs	Discopexy using suture bone anchors	Discopexy using suture anchors seemed to ↓ pain and ↑ mouth opening.
Xu J et al. (2023) [75]	China	12 RCTs	Intra-articular infiltration: HA vs. PRP vs. PRF	Efficacy PRP = PRF in short-term. PRF > PRP in long-term. Therefore, PRF was recommended for treating TMDs.
Ren H et al. (2022) [103]	China	27 RCTs	LLLT vs. TENS	The results of the meta-analysis showed that LLLT had better short-term efficacy than TENS in the treatment of pain caused by TMDs. Better results can be achieved with higher wavelengths. Therefore, it is recommended to treat TMDs using LLLT with a wavelength ranging from 910 nm to 1100 nm.
Srinivasulu Y et al. (2020) [101]	India	15 RCTs	AMT vs. DLX vs. PGB	The drugs evaluated showed benefits for pain reduction in patients with fibromyalgia.
Nagori SA et al. (2021) [61]	India	12 RCTs 1 R	Single-puncture vs. standard double-puncture techniques for arthrocentesis	No difference in pain or MMO with single- or double-puncture techniques for arthrocentesis.
Ávila-Curiel BX et al. (2020) [54]	Mexico	8 RCTs	Acupuncture Ozone therapy PRP Phonophoresis	Acupuncture, ozone therapy, platelet-rich plasma, or phonophoresis reported positive results in the control of pain for TMJ.
Gonzalez LV et al. (2023) [131]	Colombia	8 CTs	Arthroscopic surgery Arthroplasty in TMJ-SC	- Arthroscopic surgery: For use on masses confined to the superior TMJ space. - Open arthroplasty: For use in cases with an extra-articular extension. - A combination of both: For use on lesions extending beyond the medial groove of the condyle.
Muresanu SA et al. (2022) [117]	Romania	4 RCTs 3 CTs	Computer-assisted or digitally constructed occlusal splints vs. conventional splint	Digitally constructed occlusal splints generated results comparable to conventional splints in TMJD treatment. Some even produced better results due to the higher accuracy of the virtual articulator and the material properties of the splint materials.
Li J et al. (2022) [62]	China	26 RCTs	Arthrocentesis + CCS injection Arthrocentesis + CCS injection + stabilization splint Arthrocentesis + NSAID + stabilization splint Arthrocentesis + opioid injection Arthrocentesis + PRP Arthrocentesis + sodium hyaluronate injection Arthrocentesis + sodium hyaluronate injection + stabilization splint Arthrocentesis + stabilization splint	Arthrocentesis + PRP was the best option to reduce pain and improve MMO
Nogueira EFC et al. (2021) [63]	Brazil	5 RCTs	Arthroscopy vs. arthrocentesis	There was no increased risk of complications with arthroscopy vs. arthrocentesis. When complications were present, they were temporary.
Michiels S et al. (2019)	Belgium	8 cohort studies 2 RCTs 1 CT	Occlusal splint, exercise therapy in TMJD with tinnitus	Low-quality evidence for a positive effect of conservative treatment on tinnitus complaints. Splint therapy + exercise treatment was the best treatment approach (↓ tinnitus severity and intensity)
Nemeth A et al. (2024) [118]	United States of America	5 RCTs	Intra-articular infiltration: PRF PRP + arthrocentesis	Adding PRF injections to standard arthrocentesis protocols (vs. arthrocentesis alone or combined with other agents like PRP): ↓ pain; ↑ mouth opening, joint function, and favorable structural changes.
Li K et al. (2024) [93]	Canada	15 RCTs	BTX-A	BTX-A is a safe and effective treatment to ↓ pain and ↑ temporomandibular muscle and joint function in muscular TMD patients. A bilateral dose of 60–100 U might be an optimal choice for treating muscular TMD pain.
La Touche R et al. (2020) [36]	Spain	6 RCTs	Cervico-craniomandibular MT vs. cervical MT	Cervical MT > placebo MT or minimal intervention in ↓ pain (moderate evidence). Cervico-craniomandibular interventions achieved a greater short-term ↓ in pain intensity and increased pain-free MMO over cervical intervention alone in TMD and headache (weak evidence).
Hidalgo Ordoñez S et al. (2021) [119]	Chile	13 cases and controls	Occlusal splint	Occlusal splints:
				- First treatment of choice for temporomandibular disorders; - Allow for muscle relaxation; - Help the positioning of the condyle in centric relation; - Mitigate pain.
				The most effective treatments are multidisciplinary.
Li F et al. (2020) [76]	China	6 RCTs	Intra-articular infiltration PRP in TMJ-OA	PRP injections > placebo injections in ↓ pain in TMJ-OA at 6 months and 12 months postinjection (level of evidence: moderate).
Maheshwari K et al. (2024) [120]	Iran	4 RCTs	Anterior repositioning splint vs. occlusal splint in DDwR	- Evidence is uncertain whether other occlusal splints reduce TMJ clicking in comparison to anterior repositioning splint. - No evidence of any difference was found between the two splints.
Lee NW et al. (2023) [37]	South Korea	12 RCTs	Chuna MT	Chuna MT had a significant effect on:
				- ↑ TMJ function and quality of life; - ↓ Pain.
				Chuna MT is safe with fewer adverse events.
Tournavitis A et al. (2023) [29]	Greece	28 RCTs	Occlusal splint CBT Counseling Hypnosis MT LLLT BTX-A Photobiomodulation	- Occlusal splint alone or combined with other therapeutic modalities was the most effective treatment option for ↓ pain in TMD patients in short term. - Low-level laser and photomodulation treatment options had a significant role in short-term pain relief.
Menéndez-Torre Á et al. (2023) [38]	Spain	17 RCTs	MT vs. dry needling in myofascial TMD	Indirect comparisons between dry needling and MT showed no significant differences in their effects on ↓ pain in patients with myofascial TMD. However, MT was the intervention with the highest probability of success in ↓ pain in the short term, followed by dry needling.
Serrano Muñoz D et al. (2023) [109]	Spain	7 RCTs	Electrical stimulation	- TENS and high-voltage current stimulation ↓ pain intensity. - No evidence of the effect of different electrical stimulation modalities on range of movement and muscle activity.
De Castro-Carletti EM et al. (2023) [11]	Brazil	43 CTs	Electrotherapy	TENS can be a supplementary technique for reducing pain in patients with mixed TMD.
Zhang L et al. (2021) [121]	China	6 RCTs	Exercise therapy vs. occlusal splint	Occlusal splint therapy vs. exercise therapy:
				- similarly effective in pain relief and improvement of mandibular movement for pain in TMD patients.
Quezada DL et al. (2024) [77]	Chile	4 RCTs	Intra-articular infiltration PRP	Intra-articular infiltrations with PRP showed effectiveness in ↓ pain and ↑ interincisal distance up to six months after their administration.
Xie Y et al. (2022) [78]	China	9 RCTs	Intra-articular infiltration of CCS vs. HA vs. PRP in TMJ-OA	Intra-articular pharmacological injections of CCS, HA, and PRP had no effect on improving temporomandibular joint pain and functional outcomes vs. placebo.
Wu X et al. (2021) [111]	China	8 RCTs	GaAlAs laser treatment	Insufficient evidence to indicate an efficacy of low-level GaAlAs laser therapy in improving TMD pain and maximal oral opening.
Asquini G et al. (2022) [39]	United Kingdom	6 RCTs	MT	Very low quality of evidence supports MT for patients with TMD for successfully ↓ pain and ↑ MMO in the mid-term. Whether MT is superior to other interventions remains unclear but it is a low-cost, conservative option.
Al-Moraissi EA et al. (2020) [31]	Yemen	48 RCTs	Non-occluding splint Hard stabilization splint Soft stabilization splint Prefabricated splint Mini-anterior splint Anterior repositioning splint Counseling with or without hard stabilization splint	All occlusal splints are probably more effective treatments for arthrogenous and myogenous TMDs vs. no treatment and non-occluding splints.
				- Patients with mainly arthrogenous TMDs: The anterior repositioning splint and counseling therapy + hard stabilization splint seems to be the most effective treatment in ↓ pain and TMJ sounds (low evidence). - Patients with mainly myogenous TMDs: Mini anterior splints may be the most effective treatment in ↓ subjective pain (very low evidence).
Chung PY et al. (2019) [79]	Taiwan	5 RCTs	Injections of PRP vs. HA vs. saline solution	- PRP injection provided adjuvant efficacy to arthrocentesis or arthroscopy in ↓ pain for temporomandibular joint osteoarthritis in the long term. - PRP injection ↓ pain better (vs. HA injection, saline injection, or no injection).
Zwiri et al. (2020) [112]	Malaysia	25 RCTs 6 nRCTs 1 R	Laser therapy	Laser therapy shows a promising outcome of pain ↓ for TMD patients.
Ramos-Herrada RM et al. (2022) [94]	Peru	8 RCTs	BTX	BTX can be used for refractory myofascial pain (in low doses in order to avoid adverse effects).
Jing G et al. (2021) [104]	China	16 RCTs	LLLT	- d1 laser therapy (energy density ranging from 0 to 10 J/cm^2^) is effective in short-term pain management of TMD patients (moderate quality evidence). - A month after treatment, the d1 laser therapy also performed better than placebo and other laser but the result did not reach statistical significance (low quality evidence).
Maximo CFGP et al. (2022) [105]	Brazil	10 RCTs	LLL photobiomodulation	Scarcity of literature regarding masticatory functions. In the intervention groups, LLL photobiomodulation had significant results, particularly in the amplitude of mouth opening.
Van der Meer HA et al. (2020) [32]	The Netherlands	5 RCTs	MT Joint and muscle exercises Counseling	Very low certainty that there is an effect of physical therapy for TMD for concomitant headache intensity vs. control.
Honnef LR et al. (2022) [122]	Brazil	10 CTs	Stabilization splints	A positive effect on signs and symptoms of TMDs of muscular origin of a stabilization splint could not be confirmed or refuted based on very low-quality evidence found.
Liu GF et al. (2021) [57]	China	10 RCTs	Warm needle acupuncture Acupuncture Drug therapy Ultrasonic therapy	Warm needle acupuncture may have a significant therapeutic effect and clinical significance for TMDs (vs. acupuncture, drug therapy, ultrasonic therapy, and electric acupuncture).
Di Francesco et al. (2024) [87]	Italy	11 RCTs	Acupuncture Laser acupuncture	- Evidence for acupuncture as a symptomatic treatment for TMD is limited. - High efficacy of laser acupuncture was reported.
Ferrillo M et al. (2022) [40]	Italy	13 RCTs	Occlusal splints LLLT MT Ozone therapy	Conservative approaches might be effective in pain relief for intracapsular TMD patients.
Zhang Y et al. (2023) [113]	China	28 RCTs	Laser therapy	Laser therapy:
				- ↓ Pain but - Small effect on improving mandibular movement.
Herrera-Valencia A et al. (2020) [41]	Spain	6 RCTs	MT MT + therapeutic exercises	MT seems to be an effective in the medium term, and the effect appears to ↓ over time. The effects of MT + therapeutic exercise can be maintained in the long term.
Zhang SH et al. (2020) [123]	China	11 RCTs	Occlusal splints	An occlusal splint can be considered especially in patients with signs and symptoms of restriction of mandibular movement and pain.
Ferrillo M et al. (2022) [42]	Italy	16 RCTs	MT Occlusal splints	Rehabilitative approaches might be effective in ↓ pain in muscle-related TMD patients.
Ruiz-Romero V et al. (2022) [102]	Spain	3 RCTs	CS GS	CS + GS is effective in symptomatic and functional improvement of TMJ in TMD (without notable adverse effects):
				- ↓ pain, inflammatory biomarkers in synovial fluid, and joint noise; - ↑ MMO.
Kulkarni S et al. (2019) [99]	Australia	11 RCTs	NSAID	NSAIDs can ↓ pain and ↑ mouth opening. Insufficient evidence to conclude the type, dosage, and duration for each diagnostic category of TMDs.
Goker F at al. (2021) [65]	Italy	26 RCTs 2 CTs 1 R	Intra-articular injections: HA + arthrocentesis	HA injections with/without arthrocentesis seems to be beneficial in terms of clinical symptoms and quality of life.
Ulmner M et al. (2024) [66]	Sweden	36 RCTs 15 Obs	Arthrocentesis vs. conservative management vs arthrocentesis + HA vs. arthroscopy + PRGF vs. arthroscopy	- Arthrocentesis performed better than conservative management. - Non-invasive management is considered the primary measure.
Idañez-Robles AM et al. (2023) [52]	Spain	16 RCTs	Therapeutic exercise	Therapeutic exercise is an effective therapy to ↓ pain and ↑ the pain pressure threshold and active and passive MMO.
Agostini F at al. (2023) [74]	Italy	13 RCTs 5 CTs	Intra-articular injections: HA	- Intra-articular HA injections has intriguing effects in ↓ pain intensity and ↑ functioning. - There is no agreement on the effectiveness of a combination of arthrocentesis or arthroscopy with HA injections.
Gutiérrez IQ et al. (2022) [67]	Spain	8 RCTs	Intra-articular injections: PRP or PRGF + arthrocentesis or arthroscopy	PRP or PRGF demonstrated slightly better clinical results but was not significantly different from that of the control group.
Rodhen RM et al. (2022) [128]	Brazil	17 nRCTs 2 RCTs	Discectomy Arthroplasty Condylotomy Eminectomy Arthroscopy Discoplasty Disc repositioning	TMJ discectomy (vs. arthroscopy, eminectomy, and discoplasty): ↓ joint pain; ↑ mouth opening. Minimally invasive surgical procedures (arthroscopy): first-line treatment option for arthrogenous TMD management.
Park EY et al. (2023) [88]	South Korea	22 RCTs	Acupuncture	Acupuncture significantly improved outcomes versus active controls and when add-on treatments were applied.
Askar H et al. (2021) [133]	United States of America	20 Rs 6 Ps 1 cross-sectional study	Arthroscopic disk repositioning vs. open disk repositioning	Both arthroscopic and open disc repositioning ↑ clinical outcomes (pain scores and maximal incisal opening).
Nagori SA et al. (2019) [68]	India	3 RCTs 2 CTs 1 R	Splint therapy + arthrocentesis	Splint therapy may not improve outcomes after arthrocentesis.
Alkhutari AS et al. (2021) [116]	Yemen	24 RTCs	Stabilization appliance vs. non-occluding appliance (active placebo)	Stabilization appliances vs. non-occluding appliances: stabilization appliances’ treatment efficacy is beyond the placebo effect.
				- No significant difference in reported pain intensity at follow-ups. - Significant difference in number of participants reporting treatment satisfaction with reduced pain, and lower number needed to treat in favor of stabilization appliances.
Liapaki et al. (2021) [80]	United States of America	9 RCTs	Intra-articular injections: HA vs. CS vs. PRP/PRGF with or without arthrocentesis in TMJ-OA	All injectables + arthrocentesis were efficient in alleviating pain and improving MMO in TMJ-OA patients.
Da Silva Mira PC et al. (2024) [106]	Brazil	4 RCTs 3 nRCTs	LLLT	LLLT may alleviate symptoms in patients with a TMD.
Ahmad SA et al. (2021) [107]	India	37 RCTs	LLLT	LLLT appears to be efficient in ↓ TMD pain. Advantages: non-invasive, reversible, with fewer adverse effects, and may also improve the psychological and emotional aspects.
Fertout A et al. (2022) [108]	France	6 RCTs 6 nRCTs 1 cross-over trial 1 CT	TENS	TENS: ↓ electrical muscular activity; ↓ thickness of the masseter muscles; ↑ function and comfort; ↓ pain.
La Touche R et al. (2022) [43]	Spain	10 RCTs	MT + therapeutic exercise in DDwoR	- Therapeutic exercise or MT may be beneficial in the treatment of disc displacement without reduction. - Limited evidence suggests that exercise significantly improves mouth opening in comparison to splints.
Liberato FM et al. (2023) [44]	Brazil	5 RCTs	MT	MT: Reduction in pain intensity; Improvement in jaw function.
De Melo LA et al. (2020) [33]	Brazil	5 RCTs	MT	MT alone is Better than no treatment; No better than BTX. MT combined with counseling is no better than counseling alone. MT combined with therapeutic exercise is better than therapeutic exercise alone.
Derwich M et al. (2021) [81]	Poland	16 RCTs	Arthrocentesis with intra-articular injections: HA vs. CCS vs. PRP in TMJ-OA	Arthrocentesis alone: Improvement in jaw function; Reduction in pain intensity. Arthrocentesis with injections of HA or CCS: No improvement in final clinical outcomes. CCS: Chondrotoxicity on articular cartilage; No better than HA or arthrocentesis alone or combined. PRP: No improvement in MMO.
El-Kahky AM et al. (2022) [95]	Egypt	20 RCTs 3 cross-over trials 13 Ps 3 Rs	BTX-A	BTX-A in the myogenous type of TMD: Effective, safe, and minimally invasive; Better than active treatments, LLLT, needling, acupuncture, and surgery.
Liu Y et al. (2020) [69]	China	11 RCTs	Intra-articular injections: HA, dexamethasone, prednisolone, betamethasone, betamethasone + HA, morphine, tramadol, PDGF Arthrocentesis combined/alone in TMJ-OA	Tramadol, morphine, and PDGF injections after arthrocentesis: Reduction in pain; Improvement in joint opening. HA: Improvement of MMO in short term CCS + HA: Reduction in symptomatology of TMJ-OA patients.
Fouda AAH et al. (2020) [124]	Egypt	22 RCTs	Stabilizing splint, Michigan splint, centric relation appliance, flat occlusal appliance, soft or hard splints, vinyl appliances, and positioning splints	Oral splints: No reduction in pain; No improvement of MMO; Placebo effect in combination with non- or minimally invasive treatments for TMJD.
Argueta-Figueroa L et al. (2022) [50]	Mexico	14 RCTs	Acupuncture, physiotherapy, LLLT, and massage	Acupuncture, physiotherapy, LLLT, and massage: Reduction in pain intensity.
Derwich M et al. (2023) [97]	Poland	8 RCTs	Oral glucosamine in TMJ-OA	Oral glucosamine: Reduction in TMJ pain in long term; Increase in MMO; Anti-inflammatory effects.
Melis M et al. (2022) [53]	Italy	4 RCTs	Oral myofunctional therapy	Oral myofunctional therapy: Effective for TMDs; Favorable cost–benefit and risk benefit ratios.
Montinaro F et al. (2022) [100]	Italy	4 RCTs	Oral NSAIDs	Oral NSAIDs: Improvement in TMJ pain; Effective first approach to control muscle and joint pain.
Riley P et al. (2020) [125]	United Kingdom	52 RCTs	Oral splints	Oral splints: No reduction in pain in TMDs; Insufficient evidence to determine whether or not splints reduce tooth wear in patients with bruxism.
Mittal N et al. (2019) [132]	India	7 RCTs 19 Rs	Gap arthroplasty vs. interpositional gap arthroplasty vs. reconstruction arthroplasty vs. distraction osteogenesis in TMJ ankylosis	Interpositional gap arthroplasty: Highest improvements in MMO.
Torres-Rosas R et al. (2023) [55]	Mexico	8 RCTs	Ozone therapy	Ozone therapy: Reduction in TMJ pain; Improvement in MMO; No better alternative than occlusal splints and pharmacotherapy.
Minervini G et al. (2024) [98]	India	8 RCTs	NSAIDs CCS Diazepam Morphine PGB AMT Gabapentin	NSAIDs: Effective in the treatment of acute pain. Opioids: Substitute for NSAIDs in the case of patients with previous gastrointestinal bleeding or in the case of acute moderate/severe TMJ pain. CCS: Used in treatment of acute moderate/severe pain; The first choice is an intra-articular injection. Myorelaxants: The drugs of choice either for acute contractions and/or contractures or are used to treat chronic pain. Antidepressants: For chronic pain and in patients refractory to bite therapy. Anticonvulsants: For neuropathic pain and thus chronic TMJ pain. Benzodiazepines: Used in treatment of chronic myofascial pain. Pharmacological treatment must be supported by functional therapy, physiotherapy, and behavioral therapy.
Christidis N et al. (2024) [82]	Sweden	40 RCTs	BTX-A NSAIDs CCS Dextrose Clonazepam Morphine 5 mg Morphine 1.5 mg Magnesium sulfate Lidocaine Melatonin Cyclobenzaprine Granisetron PRP	For muscular TMDs, the best drugs are BTX-A, granisetron, PRP, and muscle relaxants. For joint TMDs-J, the best pharmacological treatment approaches are NSAIDs, CCS, HA, and dextrose.
Pimentel de França AM et al. (2021) [114]	Brazil	12 RCTs	Photobiomodulation	Photobiomodulation: Reduction in pain intensity; Complicated standardization guidelines; No clear effects on TMJ mobility and function.
Al-Hamed FS et al. (2021) [86]	Canada	9 RCTs	Intra-articular injections: platelet concentrates vs. HA vs. saline solution	Platelet concentrates: Reduction in pain when compared to HA during the first 3 months after treatment; Reduction in pain and increase in MMO for longer durations when compared to saline solution.
Haddad C et al. (2023) [70]	Lebanon	5 RCTs	Intra-articular injections: PRP vs. HA vs. saline solution after arthrocentesis	PRP injections: Improvements in mandibular range of motion and pain intensity up to 12 months after treatment.
Penlington C et al. (2022) [27]	United Kingdom	22 RCTs	CBT BT ACT	CBT: Greater reduction in pain intensity than alternative treatments at longest follow-up; Better than alternative treatments for reducing psychological distress at treatment completion and follow-up.
Siewert-Gutowska M et al. (2023) [71]	Poland	25 RCTs	Arthrocentesis	Arthrocentesis: Reduction in pain; Increase in MMO in DDwR/DDwoR. Additional intra-articular injections: HA, dexamethasone, and PRP/PRP do not improve the outcome of arthrocentesis. Intra-articular injections with medications without arthrocentesis is less effective.
Lima FGGP et al. (2024) [126]	Brazil	6 Ps	Prosthetic total joint replacement	TMJ total prosthesis is apparently a safe procedure with a high survival rate.
Peixoto KO et al. (2023) [89]	Brazil	6 RCTs	Traditional acupuncture vs. laser acupuncture	Traditional and laser acupuncture: Improvement in pain and MMO.
González-Sánchez B et al. (2023) [45]	Spain	15 RCTs	Physiotherapy	Therapeutic exercise protocols + MT are the most commonly utilized method for addressing TMDs and thus provide the best results.
Yaseen M et al. (2021) [127]	United States of America	13 Ps 4 Rs	Prosthetic total joint replacement	Prosthetic total joint replacement: Improvement in pain and MMO.
Farshidfar N et al. (2023) [115]	Iran	40 RCTs	Photobiomodulation	Photobiomodulation: Reduction in pain; Improvement in MMO. The infrared diode laser is the best option.
Lam AC et al. (2023) [46]	United States	8 RCTs	MT	Upper cervical spine MT presents limited benefits for TMDs.
Saini RS et al. (2024) [96]	Saudi Arabia	14 RCTs	BTX	BTX was not associated with better pain reduction adverse events, MMO, bruxism events, and maximum occlusal force.
Mohamad N et al. (2024) [90]	Canada	37 RCTs 15 CTs	Acupuncture	Acupuncture: Reduction in pain intensity in myogenous TMDs; Reduction in tenderness in the medial pterygoid muscle; Reduction in joint dysfunction.
Al-Moraissi EA et al. (2020) [51]	Yemen	36 RCTs	Muscle exercises + occlusal splint therapy Occlusal splint therapy Intra-articular injection of HA or CCS Arthrocentesis with or without HA, CCS, and PRP Arthroscopy with or without HA and PRP Open joint surgery Physiotherapy.	Arthrocentesis + intra-articular injections of adjuvant pharmacological agents (PRP, HA, or CCS): Pain reduction; MMO improvement. In short term: ≤5 months. In intermediate term: 6 months–4 years.
Al-Moraissi EA et al. (2022) [34]	Yemen	52 RCTs	Counseling therapy Occlusal appliances MT Laser therapy Dry needling Intramuscular injection of local anesthesia or BTX-A Muscle relaxants Hypnosis/relaxation Oxidative ozone therapy	MT is considered the most effective treatment for muscular TMDs, followed by counseling treatment, intramuscular injection of local anesthesia, and occlusal appliances.
Feng J et al. (2019) [47]	China	12 RCTs	Occlusal splint Physiotherapy Acupuncture TENS Gabapentin MT BTX-A NSAIDs Hypnosis Therapeutic exercises	Complementary therapies are more effective than placebo in reducing TMJ pain.
Li DTS et al. (2021) [72]	Hong Kong	8 RCTs 3 Ps	Arthrocentesis as the initial treatment vs. early arthrocentesis vs. late arthrocentesis	Regardless of start time, arthrocentesis results in an improvement in MMO and pain reduction. Arthrocentesis performed within 3 months of conservative treatment might produce beneficial results.
Chęciński M et al. (2022) [56]	Poland	52 RCTs	Intra-articular injections: HA CCS PRGF PRF PRP Morphine Dextrose + lidocaine Tramadol Ozone gas Bone marrow Adipose tissue	Better effects of intra-articular administration are achieved by preceding the injection with arthrocentesis. The most promising substances appear to be bone marrow and adipose tissue.
Abrahamsson H et al. (2020) [73]	Sweden	8 RCTs	Conventional repositioning Wrist pivot method Injections: Dextrose Autologous blood	Autologous blood injection into the superior joint space and pericapsular tissues with intermaxillary fixation seems to be the treatment for recurrent TMJ luxation.
Al-Moraissi EA et al. (2024) [48]	Yemen	20 RCTs	Occlusal splints LLLT MT Arthrocentesis Arthrocentesis + intra-articular injection of PRP or HA Arthrocentesis + occlusal splint	Arthrocentesis with intra-articular injection of PRP/HA: The most effective treatment in terms of pain reduction. LLLT: The best choice for increasing MMO for patients with DDwR.
Dinsdale A et al. (2022) [49]	Australia	10 RCTs 1 prepost study	Occlusal splints Photobiomodulation Needling Exercise MT Patient education	MT, needling, oral splinting, exercise, and photobiomodulation: Improvement in bite function in TMDs. Patient education: No improvement in bite function.
López JP et al. (2024A) [129]	Colombia	12 N/R	Arthroscopic discopexy: Non-rigid Semi-rigid Rigid.	Semi-rigid technique shows the best results in terms of improvement in MMO and pain reduction.
López JP et al. (2024B) [84]	Colombia	4 RCTs 1 case series	Arthroscopy + intra-articular injections: HA CCS NSAIDs PRP PRGF Sodium hyaluronate	The benefit of substances like ATM arthroscopic adjuvants has not been clearly established.

BTX, botulinum toxin; BTX-A, botulinum toxin type A; P, prospective study; R, retrospective study; RCTs, randomized clinical trials; CTs, clinical trials; nRCTs, non-randomized clinical trials; MMO, maximum open mouth; TMDs, temporomandibular disorders; TMJ, temporomandibular joint; HA, hyaluronic acid; PRF, platelet-rich fibrin; PRP, platelet-rich plasma; LLLT, low-level laser therapy (LLLT); LLL, low-level laser; TENS, transcutaneous electric nerve stimulation; nm, nanometers; AMT, amitriptyline; DLX, duloxetine; PGB, pregabalin; TMJ-SC, temporomandibular joint synovial chondromatosis; MT, manual therapy; TMJ-OA, temporomandibular joint osteoarthritis; DDwR, disc displacement with reduction; DDwoR, disc displacement without reduction; CBT, cognitive–behavioral treatment; BT, behavior therapy; ACT, acceptance and commitment therapy; CCS, corticosteroid; GaAlAs, low-level gallium aluminum arsenide; CS, chondroitin sulfate; GS, glucosamine; NSAID, nonsteroidal anti-inflammatory drug; Obs, observational study; PRGF, plasma rich in growth factors; PDGF, platelet-derived growth factor; N/R, not reported; ↑ increased or improved; ↓ decreased or reduced.

## Data Availability

The raw data supporting the conclusions of this article will be made available by the authors on request.
